# HEAD AND NECK COOLING AS AN EMERGENCY DEPARTMENT THERAPY TO DECREASE PROGRESSION OF CONCUSSIVE SYMPTOMS: A PILOT STUDY

**DOI:** 10.51894/001c.123017

**Published:** 2024-08-30

**Authors:** Daniel Mattox

**Affiliations:** 1 Emergency Medicine Corewell Health Lakeland

## 53

### INTRODUCTION

Mild traumatic brain injury (TBI) accounts for 700,000 emergency department (ED) visits annually in the United States. Headache, vision changes, dizziness, nausea and vomiting, cognitive dysfunction and changes in mood are all common.

### OBJECTIVES

Head and neck cooling has been shown in some athletic populations to be helpful, but it is unknown if it could be a useful therapy in the emergency department. Our goal was to evaluate the degree of symptomatology and improvement in an emergency department to better estimate the numbers needed for a wider trial.

### METHODS

IRB approval was obtained. Informed Consent was obtained by the research team when qualifying patients presented during the team’s availability. Inclusion criteria included awake non-pregnant adults with head injury symptoms presenting within 24 hours of arrival. Exclusion criteria included repeat emesis, known intracranial pathology, and scalp laceration. On even dates patients were treated with a cooling helmet (provided by Catalyst Cryohelmet) for 30 minutes, on odd dates they were not. Their symptoms were evaluated using the validated Post-Concussion Symptom Severity Score Index (PCSSI) at zero and one hour in the ED.

### RESULTS

Eighteen patients (10 women, 8 men) agreed to participate with mean age of 47. Seven patients were randomized to the helmet group. Their PCSSI improved by 12.3, from 31.5 to 19.3 (paired t-test p-value = 0.11, 95% CI -3.85 to 28.42). Eleven patients were randomized to the control group. Their PCSSI improved by 8.9, from 58.2 to 49.3 (paired t-test p-value = 0.039, 95% CI 0.56 to 17.25). Due to the wide range of presenting symptoms about 250 people per group would be needed to demonstrate a 5-point greater improvement in the treatment group (if PCSSI was 40 +/- 20). With a narrower range of symptoms (PCSSI 40 +/- 10) then 60 patients per group could demonstrate that difference.

### CONCLUSIONS

Both patients treated with and without the cooling helmet demonstrated improvement, however this was statistically significant only in the larger control group. A larger study would require a much narrower range of presenting symptoms and should likely focus on the more symptomatic patients to achieve this outcome.

